# Immunogenicity after Second ChAdOx1 nCoV-19 (AZD1222) Vaccination According to the Individual Reactogenicity, Health Status and Lifestyle

**DOI:** 10.3390/vaccines9121473

**Published:** 2021-12-13

**Authors:** Hyunji Choi, Sun-Min Lee, Seungjin Lim, Kyung-Hwa Shin, Taeyun Kim, Won-joo Kim, Misook Yun, Seung-Hwan Oh

**Affiliations:** 1 Department of Laboratory Medicine, Pusan National University Yangsan Hospital, Yangsan 50612, Korea; woojukirin@naver.com (H.C.); sweethome01@naver.com (W.-j.K.); paracelsus@hanmail.net (S.-H.O.); 2 Department of Laboratory Medicine, Pusan National University School of Medicine, Yangsan 50612, Korea; skh2009pnuh@gmail.com; 3 Division of Infectious Diseases, Department of Internal Medicine, Pusan National University Yangsan Hospital, Yangsan 50612, Korea; babopm@naver.com; 4 Department of Laboratory Medicine, Pusan National University Hospital, Busan 49241, Korea; 5 Department of Internal Medicine, The Armed Forces Goyang Hospital, Goyang 10271, Korea; jimsb89@naver.com; 6 Division of Biostatistics, Research Institute for Convergence of Biomedical Science and Technology, Pusan National University Yangsan Hospital, Yangsan 50612, Korea; msyun@pusan.ac.kr

**Keywords:** SARS-CoV-2, vaccines, adverse effect, neutralizing antibody, cellular immune response, BMI

## Abstract

The immune-acquired responses after vaccination vary depending on the type of vaccine and the individual. The purpose of this study was to investigate the relationship between the acquisition of immunity and the side effects, health status, and lifestyle after completion of the second dose of AZD1222. Blood samples were collected after a second dose of AZD1222. The Euroimmun Anti-SARS-CoV-2 ELISA (IgG) for anti-S1 antibody, the cPASS SARS-CoV-2 neutralizing antibody detection kit for the surrogate virus neutralization test, and the T-spot Discovery SARS-CoV-2 kit were used to identify cellular immunogenicity. Patient experience of adverse effects was investigated using questionnaires. Information on health status and lifestyle were collected from the most recent health checkup data. Generally, females experience more reactogenicity in both intensity and duration. The rash of the first shot and chills of the second shot were associated with humoral immunity. However, comprehensive adverse effects had no correlation with humoral and cellular immunity. The T-spot-positive group had a higher creatinine level, which reflects muscle mass, than the T-spot-negative group. Males presented a higher level of T-spot assays. Body mass index and age were negatively correlated with the T-spot assay and anti-S1 antibody, respectively. Immune acquisition after the second AZD1222 shot was not associated with reactogenicity. However, individuals’ sex, age, and BMI were found to be associated with immunogenicity after vaccination.

## 1. Introduction

Elucidating the immune responses following vaccination against Severe Acute Respiratory Syndrome Coronavirus 2 (SARS-CoV-2) is of particular importance. Responses may vary according to the type of vaccine, and interindividual variability may also exist [1]. Along with demonstrating the efficacy of vaccines in preventing symptomatic disease and hospitalization through several clinical trials, disentangling the underpinnings of humoral and cellular immunity related to SARS-CoV-2 exposure has also been progressing [2]. For example, neutralizing antibody (nAb), in response to virus invasion, protects host cells by binding to the virus receptor-binding protein as a part of the humoral response of the adaptive immune system [3]. In addition, SARS-CoV-2-specific memory T cells are likely to provide long-term immune protection [4].

ChAdOx1 nCoV-19 (AZD1222) is an adenovirus-vectored SARS-CoV-2 vaccine that mediates the production of SARS-CoV-2 spike glycoprotein in the human body, consequently inducing humoral and cellular immunity to SARS-CoV-2 [5]. The recommended interval between the first and second doses was 4 to 12 weeks, and the overall efficacy was 74.0%; in the group aged >65 years, the efficacy was 83.5% [6]. 

Adverse events (AEs) following AZD1222 vaccination are of concern, with 61–97% of people receiving the vaccine presenting local side effects, such as pain or redness at the injection site, and 65–98.9% presenting systemic side effects, such as fever, tiredness, and muscle pain [6,7,8]. It is unclear whether antigen-specific immune acquisition is independent of AEs [9]. People may refuse to be vaccinated, if the discomfort from side effects of the vaccine outweigh the benefits, especially in countries or age groups where the incidence of severe disease is low [10,11]. 

Individual health status and lifestyle have been studied in relation to their influence on the immunogenicity of various vaccinations [12]. Factors such as sex, age, and innate immunity are beyond our control, but certain health conditions or lifestyle factors, such as obesity, physical activity, muscle exercise, alcohol consumption and smoking, can be controlled if they have a positive effect on immunogenicity.

Although several studies have evaluated the relationship between reactogenicity and immunogenicity following AZD1222 vaccination, the immune-related profiles of fully vaccinated participants were not evaluated [13,14,15,16], nor were individualized approaches based on health status performed [12]. In this context, the present study aimed to evaluate the association between reactogenicity and immunogenicity from the perspective of nAb, which may provide immediate protection against the virus, and memory T cells, which may reflect immunological memory, in fully vaccinated healthcare workers (HCWs), who measure annually individual health status and lifestyle.

## 2. Materials and Methods

### 2.1. Study Participants

A total of 80 fully vaccinated HCWs from the Pusan National University Yangsan Hospital (PNUYH) were recruited 2 weeks after their second shot of AZD1222. Surveys and blood sampling were conducted from 15 June 2021 to 1 July 2021. One participant who tested positive for Euroimmun Anti-SARS-CoV-2 nucleocapsid protein (NCP) enzyme-linked immunosorbent assay (ELISA) (IgG), which indicates a previous history of SARS-CoV-2 infection, was excluded. The most recent health checkup data were collected to identify individual health conditions. The aspirated blood samples were divided into 8 mL heparin tubes for T-spot tests, and 5 mL SST tubes for antibody and nAb tests. The T-spot tests were completed within 24 h after aspiration. The samples for the antibody test were divided into three aliquots after centrifugation and were frozen, then thawed for examination.

### 2.2. Measurement of Adverse Events

AEs following the first and second injections were investigated using questionnaires and evaluated in terms of the severity and the intensity. The AEs identified included four local and eight systemic outcomes. Local side effects included tenderness, pain, redness, and swelling. Systemic side effects included fever, chills, headache, myalgia (muscle pain), fatigue, nausea and vomiting, rash, and anaphylaxis (or anaphylactoid reaction). All participants were interviewed about their questionnaires by laboratory physicians and special circumstances, such as outpatient visits to hospitals and hospitalizations, were identified. The intensity of the AEs was subjectively graded from 0 to 4:0 (none), 1 (mild; did not affect daily or occupational activities), 2 (moderate; affected daily or occupational activities), 3 (severe; daily or occupational activities impossible), and 4 (very severe; life-threatening, admission, or death), based on the Food and Drug Administration’s guidance for toxicity grading scales for vaccines [17]. In addition, one point was added if the individual experienced a high fever (≥39 °C) or vomited more than twice. Finally, to integrate the severity and the duration of the AEs, the sum of reactogenicity intensity (SRI) and sum of reactogenicity intensity and duration (SRID) were calculated using the following formula [15].

$$\mathrm{SRI}=\sum \left(Ijr\right)$$

SRID=∑(Ijr×Djr)

where *I* = the intensity of AEs, *D* = the duration (days) of AEs, *j* = the dose of the vaccine (*j* = 1 or 2), and *r* = AEs (four local and eight systemic AEs).

### 2.3. Measurements of Individual Health Status

The most recent health checkup data collected within one year were used to determine individual health status. The health checkup data included anthropometric profiles; body mass index (BMI) and waist circumference (WC), existence of comorbidity, smoking status, alcohol consumption, physical activity, muscle exercise, blood pressure (BP), and laboratory studies; hemoglobin (Hb), fasting blood glucose (FBG), serum creatinine (Cr), estimated glomerular filtration rate (eGFR), alanine transaminase (AST), aspartate transaminase (ALT), gamma-glutamyl transferase (GGT), triglycerides (TG), high-density lipoprotein cholesterol (HDL-C), and low-density lipoprotein cholesterol (LDL-C) levels) and total cholesterol (TC). Smoking status was classified according to the national health interview survey of the United States [18]. Nonsmokers and former smokers were designated to the ‘No’ smoking category and current smokers were designated to the ‘Yes’ category. Level of alcohol consumption was calculated following the Korean alcohol guidelines [19]. In terms of physical activity and muscle exercise, the global physical activity questionnaire was used [20]. The judgment criteria were in accordance with the guidelines of the Ministry of Health and Welfare of Korea [21]. Adequate physical activity was defined as more than 150 min per week of moderate activity (activity causing a slight shortness of breath or increase in heart rate), while adequate muscle exercise was defined as carrying out exercises such as push-ups, sit-ups, dumbbells presses, etc., more than twice per week [21]. 

### 2.4. Measurement of Cellular and Humoral Immune Responses

#### 2.4.1. Humoral Immunity

The Euroimmun ELISA (Euroimmun, Lübeck, Germany) was used to semi-quantitatively detect IgG antibodies against the viral S1 protein. The Euroimmun Anti-SARS-CoV-2 NCP ELISA (IgG) test was used to exclude participants who had experienced an actual infection, because AZD1222 does not include NCP as a viral antigen. Both tests were performed using thawed aliquots, and for the signal to cut-off (S/Co) value, dividing the optical density (OD) of the sample by the OD of the calibrator, ≥1.1 was determined to be positive.

The plaque reduction neutralization test (PRNT), a conventional virus neutralization test (cVNT), is considered the gold standard to detect neutralizing antibodies. However, because the method requires a biosafety level (BSL)-3 facility, it is not easily accessible in general laboratory conditions, and has low test efficiency. Therefore, we utilized the cPASS SARS-CoV-2 neutralizing antibody detection kit, surrogate VNT, sVNT (Genscript Biotech Corporation, Piscataway, NJ, USA), which is known to have a high correlation with the PRNT [22,23].

#### 2.4.2. Cellular Immunity

The T-spot Discovery SARS-CoV-2 kit (Oxford Immunotec Ltd., Abingdon, Oxfordshire, UK) was used to confirm cellular immunity. This method is a simplified variant of the ELISpot assay technique. Peripheral blood mononuclear cells (PMBCs) are isolated from a whole blood sample. Subsequently, in response to SARS-CoV-2 antigens, interferon-gamma (IFN-gamma) is secreted from the PMBCs and forms spots which are measured. Two experts in laboratory medicine read the report and averaged the results, with the difference in the number of spots read between the two readers not exceeding three. The T-spot Discovery SARS-CoV-2 kit is composed of four panels: panel 1, panel 3, panel 4, and panel 13, consisting of the SARS-CoV-2 S antigen, nucleocapsid protein, membrane protein, and the high-homology regions of the coronaviruses, respectively. For the quantitative determination of the T-spot, the value obtained by subtracting the number of nil control spots from the number of panel 1 (S antigen) spots was considered the index. Although the T-spot Discovery SARS-CoV-2 kit did not provide a cut-off point, we considered 10 spots/250,000 PBMCs or more to be positive, as suggested in a previous study that defined the cut-off point as the number of spots of mean + 3 standard deviations of the uninfected and unvaccinated population [22]. 

### 2.5. Statistical Analysis

Student’s *t*-test, one-way ANOVA, and the chi-square test were used to compare two and three continuous and nominal variables, respectively. For post hoc analysis, a Tukey’s test was used to identify any significant association between two variables. A linear regression analysis was used to reveal the association between anti-S1 antibody and sVNT. Several variables which were considered meaningful for immunogenicity or presented a slight association with immune acquisition in the Student’s *t*-test (sex, age, BMI, muscle exercise, SRI, and SRID) were selected for multivariate analysis. The univariate model was conducted to select variables and multivariate analysis with backward selection was applied for anti-S1 antibody, T-spot, and sVNT. All statistical analyses were performed using IBM SPSS Statistics for Windows, version 25.0 (IBM Corp., Armonk, NY, USA). A *p*-value < 0.05 was considered statistically significant, and a *p*-value < 0.10 was considered to indicate borderline significance.

## 3. Results

### 3.1. Characteristics of the Study Participants

The participants included 21 men and 58 women. The mean ages of the men and women were 40.4 (range: 29–60) and 35.5 (range: 20–51), respectively (Table 1). The average number of days the blood sample was taken after the second injection was 15.7 days and the interval between the first and second shots was 78.6 days. The male participants were on average older than the female participants. Systolic and diastolic BP, BMI, WC, Hb, FBG, serum Cr, GGT, TC, HDL-C, LDL-C, and TG levels were higher in the male than female participants. Four, three, three and one HCWs had been previously diagnosed as pulmonary tuberculosis, hypertension, dyslipidemia and diabetes mellitus, respectively. Two of them had more than two comorbidities.

### 3.2. Humoral and Cellular Immunogenicity

**Figure 1:**
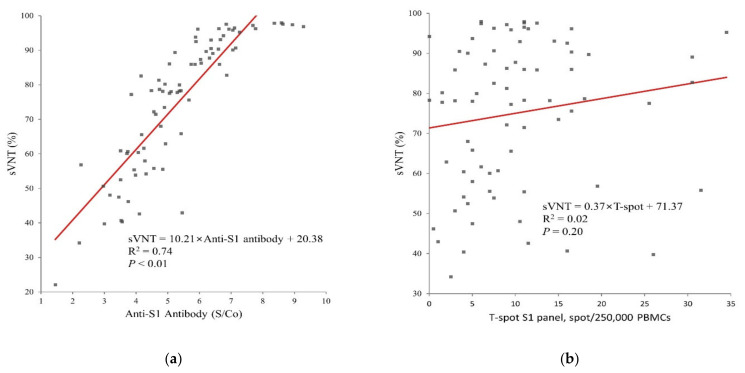
Relationship between the immunogenicity assays in 79 participants who are full vaccinated with AZD1222 (**a**) value of S/Co of anti-S1 antibody and sVNT (%) (**b**) Spots of Discovery SARS-CoV-2 kit S1 panel and sVNT. S/Co; signal to cut-off, sVNT; surrogate virus neutralization test.

**Figure 2:**
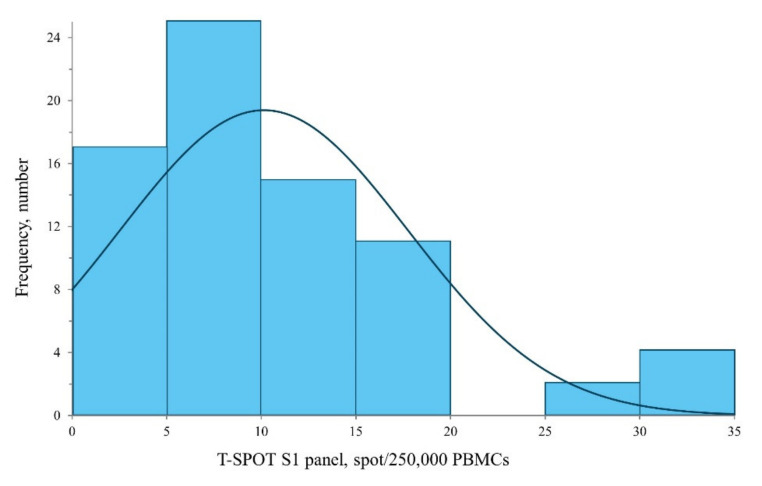
Distribution of T-spot assay results in 74 participants who completed second AZD1222 vaccination with its Gaussian density estimation line.

Fully vaccinated participants returned 100% positive results for anti-S1 antibody and 98.7% positive for sVNT (Table 1). A strong correlation between anti-S1 antibody and sVNT was observed (R^2^ = 0.743, *p* < 0.001, Figure 1a), and there was no significant correlation between sVNT and T-spot (Figure 1b). The median T-spot value among participants with full vaccination was 9 spots/250,000 PMBCs (interquartile range: 5.0–12.6, Figure 2).

In terms of cellular immunity, 32 of participants (43.2%) presented as positive in the T-spot test. The T-spot-positive group showed a higher Cr and lower eGFR (Table 2); however, in both groups the values of eGFR were in the normal range, except for one female participant.

### 3.3. Reactogenicity

**Figure 3:**
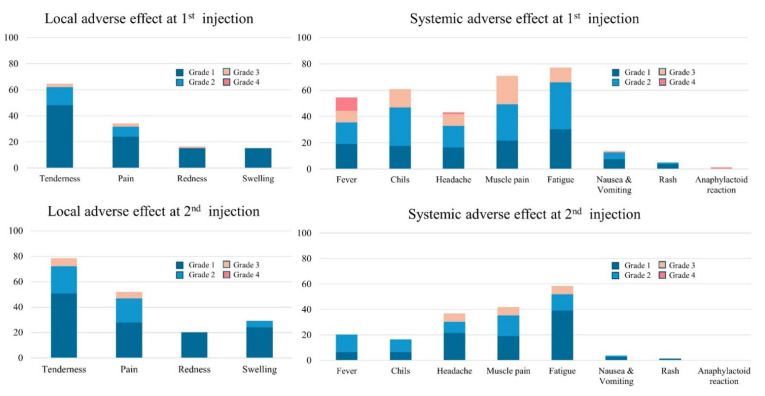
Systemic and local adverse events during first and second injections according to the grade of intensity.

SRI was significantly higher in women than in men, and SRID was also higher in women than in men (Table 1). Data are presented as mean ± standard deviation for continuous variables and numbers with percentages for categorical variables, otherwise stated. sVNT; surrogate virus neutralization test, SBP; systolic blood pressure, DBP; diastolic blood pressure, BMI; body mass index, WC; waist circumference, Hb; hemoglobin, FBG; fasting blood glucose, Cr; creatinine, eGFR; estimated glomerular filtration rate, AST; aspartate aminotransferase, ALT; alanine aminotransferase, GGT; gamma-glutamyl Transferase, TC; total cholesterol, HDL-C; high-density lipoprotein cholesterol, LDL-C; low-density lipoprotein cholesterol, TG; Triglyceride, SRI; sum of reactogenicity intensity, SRID; sum of reactogenicity intensity and duration. The most frequently experienced systemic AE was fatigue, after both the first injection (77.2%) and the second (58.2%) (Figure 3), followed by myalgia with 70.9% after the first and 41.8% after the second shot. One participant had an anaphylactoid reaction of swelling of the lips after the first injection and received outpatient management for 10 days. The most common local AE was tenderness after both the first and second injections (64.6% and 78.5%, respectively). Compared to the first injection, after the second injection the proportion of systemic AEs decreased, but the rate of local AEs increased. 

### 3.4. Association between Immunogenicity, Reactogenicity, and Individual Health Status

In the comparison between T-spot-positive and -negative groups, the T-spot-positive group presented higher Cr levels and lower eGFR values (Table 2). Associations between anti-S1 antibody, sVNT, and the T-spot level with the individual AE categories were additionally identified (Table A1). The experience of a rash following the first shot was associated with the sVNT level (*p* = 0.02), and the experience of chills after the second shot was associated with the levels of anti-S1-Ab and sVNT (*p* = 0.03 in both cases). Tenderness after the first shot showed a mild correlation with sVNT (*p* = 0.09).

## 4. Discussion

Some individual health status factors, including lifestyle, were found to be meaningful (Table A2). The participants’ BMI and grade of muscle exercise had borderline significance with the T-spot test results (*p* = 0.08 and 0.09, respectively). Statistical significance was not achieved for the association between other individual health factors and lifestyle and immunogenicity after the completion of AZD1222 vaccination. 

In multivariate analyses, male sex was positively associated with the value of the T-spot assay, and BMI was negatively associated with it (Table 3). For humoral immunity, age was slightly negatively associated with anti-S1 antibody and sVNT.

As of 12 September 2021, a total of 5,534,977,637 vaccine doses had been administered worldwide, according to the World Health Organization (WHO) dashboard. After the WHO listed AZD1222 for emergency use in February 2021, nearly 67 million doses in the European Union and 21 million doses in South Korea had been administered by September 2021. However, a global imbalance of the vaccine supply has left some people without access. For example, only around 3% of Africans are fully vaccinated, while more than half of people in the United States have completed vaccination. In addition, to make matters worse, the SARS-CoV-2 Delta variant has surged worldwide. Many people have not yet been vaccinated. Furthermore, since antibody levels decrease over time after vaccination [23], additional vaccinations are being considered in each country. Studies on the factors that can increase immunity during vaccination are insufficient.

The present study assessed the relationship between immunogenicity and reactogenicity in healthy HCWs who received AZD1222 in a tertiary hospital, not only evaluating the immunogenicity in terms of both the humoral and cellular aspects, but also considering individual health status as an important contributor to immune acquisition. We identified that comprehensive AEs are not correlated with anti-S1 antibody, sVNT, or T-spots. Creatinine was found to be at a higher level in the T-spot-positive group compared with the T-spot-negative group. Age was correlated with humoral immune acquisition, and sex and BMI were correlated with the value of the T-spot assays. The results from our study contribute to the literature regarding immunogenicity and reactogenicity in people receiving AZD1222.

Various laboratory-based tests have been developed to measure immunogenicity, or the ability of cells/tissues to provoke immune responses to specific antigens, proteins, or peptide drugs, at an individual level [23]. Immunogenicity can be measured considering the aspects of humoral and cellular immunity, using the nAb level and T-cell activity, respectively [2]. Neutralizing antibodies are responsible for the initial protection against viral infections by binding outside the virus, while cellular immunity plays an important role in clearing virus infected host cells [24]. Inter-individual variability in the immune system; the combination and strength of cellular and humoral immunity, might result in the diversity of vaccine efficacy. In our study, the humoral immunity acquisition was almost 100% after the second dose. However, the cellular immunity response was variable.

In severe SARS-CoV-2 infection, the T-cell response is decreased compared with the average SARS-CoV-2 infection in early infection, implying cellular immunity is correlated with disease severity and prognosis [24,25]. Neutralizing antibody seems to be a protective factor, and T-cell response offers the possibility of protection through reducing viral replication [26]. Concerning efficacy related to disease severity, studies on cellular immunity acquisition factors are needed.

We found a positive linear correlation between nAb and sVNT, which could be explained by nAb being a part of the anti-S1 antibody [14,15,27]. In our study, humoral and cellular immunity were found to be independent (Figure 1b), and this is in line with previous results [5,23,24]. 

Reactogenicity refers to a subset of physical manifestations of the inflammatory response after vaccination. Although a few studies have evaluated the effect of reactogenicity on immune acquisition, the results have been variable, and did not include fully vaccinated participants [13]. For example, one study showed no association between reactogenicity and immunogenicity, and another found only a weak correlation between AEs and immune acquisition after the first shot [15]. We identified a rash after the first dose and chills after the second dose as related to immunogenicity, but SRI and SRID, which are considered in the full spectrum of AEs, showed no association with construction of an immune response. This might be attributable to a prime-boost regimen improving immunogenicity in a more strengthening and uniform way than a single shot [5,14]. 

Creatinine level has been considered to be a surrogate marker for muscle mass [28], and several studies have revealed the relationship between muscle mass and immunity in old age [29,30]. Interleukin-6 is known to be associated with the frailty of the immune system, and has a tendency to increase in people with a lower muscle mass and strength [29]. The toll-like receptor signaling pathway produces cytokines that affect vaccine immunogenicity and is associated with muscle characteristics such as muscle mass and strength in old age [30]. In our study, the T-spot-positive group had a higher creatinine level. 

In a multivariate model for humoral immunity, only age was related to anti-S1 antibodies, while for cellular immunity, sex and BMI were related factors. Sex disparities in the relationship between AEs and immunogenicity may exist, with a higher likelihood of experiencing AEs in women than in men [15,31]. Hormonal, genetic, microbiome, and environmental factors may influence sex differences in immune profiles related to the outcome of vaccination, eliciting much higher levels of humoral and cellular immune responses in women than in men [31,32]. Although we found higher levels of anti-S1 antibody and sVNT in female than in male participants, statistical significance was not reached. However, T-spots showed higher levels in male. Generally, females have higher antibody levels, B-cell numbers, and CD4 T-cell cumbers, while males have higher CD8-positive T-cell counts [31,33]. However, both CD4-positive T cells and CD8-positive T cells are more active in females [31,33] and T-spots only count the T-cell number. Therefore, despite the result of the T-spot assay being higher in males, the overall cellular immunity might not be greater in males than females.

Age has been considered a relevant variable in various vaccines [24,34]. In an older population, the antibody cellular response decreased following vaccines for diphtheria, hepatitis B, hepatitis A, tetanus, and so on [12]. The older the person receiving the vaccine, the less the cellular immune response evoked by a vaccine for influenza [12]. For the SARS-CoV-2 vaccine, the humoral immune acquisition has been shown to be lower in the older population [5]. We found an association between age and humoral immunity, despite the narrow distribution of the participants’ ages (median: 36, IQR: 33–40, and range: 20–65).

Considering the paucity of data on the effect of individual health status on immunogenicity and reactogenicity, several behavior health-related markers have been introduced to evaluate potential associations [12]. In our study, BMI was considered to be a probable candidate factor in cellular immunity (*p* = 0.08) and had negative significance for T-spots in the multivariate analysis. In studies on various vaccines, BMI was found to be a possible factor in immune acquisition [12,35]. Several studies found that in an obese population, a prolonged pro-inflammatory state confused the immune response at the time of vaccination, so immune acquisition decreased compared to the non-obese population. A difference in the intestinal flora between the obese and non-obese populations may also affect the immune response [35]. 

Regular and proper exercise is protective against physical and psychological illness, affecting innate immunity by reducing chronic and acute inflammation, and consequently affecting immune acquisition after vaccination, especially in the elderly population [36,37]. However, the ideal type and intensity of exercise for increased immune acquisition has not yet been established [38]. In our study, the level of muscle exercise seemed to have a mild correlation with the T-spot results (*p* = 0.09).

Antipyretics are widely used for symptom control, and the timing of antipyretic administration is considered to be one factor associated with antibody response, as some antipyretics reduce blastogenesis [9,39]. However, in our study, there was no difference between the antipyretic use group and non-use group, and administration timing seemed to have no association with humoral and cellular immunogenicity (Table A2), similar to other studies on the antibody response to AZD1222 [14,15].

Our study has several limitations. First, the baseline value of neutralizing antibodies and T-spot assay and the level after the first shot were not measured; thus, a comparison between baseline, after first shot, and after second shot was not possible. Second, long-term analysis after the injections was not performed. Further evaluation with analysis of the long-term effects of AZD1222 on immunogenicity and its associated factors is needed. Third, considering the data of reactogenicity were collected using questionnaires, recall bias could exist. In addition, information on nutritional status and micronutrients of the lifestyles that can influence the immune status was not included in the health checkup data and was consequently impossible to be examined [12]. Fourth, our study included a relatively small number of 80 participants, and one was excluded due to previous SARS-CoV-2 infection. The age range of the participants was relatively young, so evaluation of older people was impossible. The protective efficacy could not be estimated because of the low prevalence of SARS-CoV-2 in this country. The most serious AE associated with AZD1222 was vaccine-induced thrombotic thrombocytopenia (VITT); another notable side-effect was vaccine-associated immune thrombocytopenic purpura (ITP), without thrombosis [40,41]. Many participants in this study were women less than 40 years old, which is a high-risk group for VITT and vaccine-associated ITP [42]; however, a personal interview with laboratory physicians revealed no cases of VITT and vaccine-associated ITP in the present study. This is thought to be because of the small sample size. However, the number of platelets, D-dimer, and anti-PF4, which are important parameters of VITT and vaccine-associated ITP, were not checked to facilitate a more accurate diagnosis [40,42].

Despite these limitations, the present study has several strengths. First, considering that only a few studies exist which have estimated humoral and cellular immunity together, our study may provide insight into immunological processes after SARS-CoV-2 vaccination. Second, to our knowledge, this is the first attempt to evaluate the association between reactogenicity and immunogenicity, considering individual health status and lifestyle factors. However, given statistical significance was not found regarding those factors, further longitudinal studies using much bigger samples should be performed.

## 5. Conclusions

Immunogenicity is the most important outcome following vaccination. Although the type, intensity, and duration of reactogenicity appear to be different among individuals, its correlations to humoral or cellular immunogenicity seem weak after the second dose of AZD1222. Meanwhile, each individual’s characteristics, such as sex, age, and BMI, may be associated with the acquisition of immunogenicity after vaccination. Since these factors are related to muscle mass, the relationship between healthy lifestyle habits and the successful acquisition of immunogenicity needs be studied in a large-scale cohort.

## Figures and Tables

**Table 1:** Characteristics of study participants according to sex and their differences on variables (n = 79).

		Male (n = 21)	Female (n = 58)	Total	*p*
Age	Age (years)	40.4 ± 7.5	35.5 ± 6.3	36.8 ± 7	0.01
Days between 1st and 2nd shot		77.8 ± 13.4	78.9 ± 8.6	78.6 ± 10.0	0.65
Days after 2nd shot		16.8 ± 4.8	15.3 ± 2.4	15.7 ± 3.2	0.20
Immunogenicity	S1 antibody (S/Co value)	5.2 ± 1.9	5.4 ± 1.5	5.3 ± 1.6	0.73
	S1 antibody positive	21/21 (100%)	58/58 (100%)	79/79 (100%)	N/A
	T-spot (spots)	12.9 ± 7.8	9.3 ± 7.4	10.1 ± 7.6	0.08
	T-spot positive	10/17 (52.9%)	22/46 (19.3%)	32/74 (43.2%)	0.14
	sVNT (%)	73.7 ± 20.9	75.4 ± 18.7	75.0 ± 19.2	0.73
	sVNT positive	20/21 (95.2%)	58/58 (100%)	78/79 (98.7%)	0.09
Blood pressure	SBP (mmHg)	123.7 ± 13.0	112.8 ± 10.1	115.7 ± 11.9	<0.01
	DBP (mmHg)	80.5 ± 8.5	72 ± 8.4	74.2 ± 9.2	<0.01
Anthropometrics	BMI (kg/m²)	26.8 ± 7.1	22.5 ± 3.3	23.7 ± 5	<0.01
	WC (cm)	77.5 ± 8.5	73.6 ± 13.1	74.7 ± 12.1	<0.01
Physical activities	Lack	9 (42.9%)	33 (56.9%)	42 (53.2%)	0.28
	Adequate	12 (57.1%)	25 (43.1%)	37 (46.8%)	
Weight training	Lack	8 (38.1%)	44 (75.9%)	52 (65.8%)	<0.01
	Adequate	13 (61.9%)	14 (24.1%)	27 (34.2%)	
Smoking	No	9/21 (42.9%)	58/58 (100%)	70/79 (88.6%)	<0.01
	Yes	12/21 (57.1%)	0/58 (0%)	9/79 (11.4%)	
Alcohol consumption	Mild to moderate	6/21 (28.6%)	39/58 (67.2%)	45/79 (57.0%)	0.02
	Heavy or binge	15/21 (71.4%)	19/58 (32.8%)	34/79 (43.0%)	
Comorbidity	Pulmonary tuberculosis	2/21 (9.5%)	2/58 (3.4%)	4/79 (5.1%)	0.28
	Hypertension	2/21 (9.5%)	1/58 (1.7%)	3/79 (3.8%)	0.11
	Dyslipidemia	2/21 (9.5%)	1/58 (1.7%)	3/79 (3.8%)	0.11
	Diabetes mellitus	1/21 (4.8%)	0/58 (0.0%)	1/79 (1.3%)	0.09
Laboratory analysis	Hb (g/dL)	15.0 ± 0.8	13.1 ± 1.05	13.6 ± 1.3	<0.01
	FBG (mg/dL)	102.2 ± 14.4	91.5 ± 13.1	94.4 ± 13.1	<0.01
	Cr (mg/dL)	0.8 ± 0.1	0.6 ± 0.1	0.7 ± 0.1	<0.01
	eGFR (mL/min/1.73 m^2^)	85.0 ± 15.7	92.6 ± 20.4	90.6 ± 19.4	0.13
	AST (IU/L)	23.7 ± 5.8	21.8 ± 13.7	22.3 ± 12.1	0.55
	ALT (IU/L)	32.4 ± 18.6	19.4 ± 31.9	22.8 ± 29.4	0.08
	GGT (IU/L)	32.5 ± 24.0	16.6 ± 11.1	20.8 ± 16.9	<0.01
	TC (mg/dL)	225.6 ± 41.9	198.1 ± 35.2	205.4 ± 38.8	<0.01
	HDL-C (mg/dL)	54.7 ± 8.8	62.1 ± 9.9	60.2 ± 10.1	<0.01
	LDL-C (mg/dL)	134.9 ± 37.5	116.3 ± 36.1	121.3 ± 35	0.04
	TG (mg/dL)	172.7 ± 82.5	98.9 ± 53.9	118.6 ± 70.3	<0.01
Reactogenicity	SIR	9.3 ± 8.5	14.9 ± 9.3	13.4 ± 9.3	0.02
	SIRD	19.1 ± 22.9	39.9 ± 32.8	34.4 ± 31.7	0.01

Data are presented as mean ± standard deviation for continuous variables and numbers with percentages for categorical variables, otherwise stated. S/Co; signal to cut-off, sVNT; surrogate virus neutralization test, SBP; systolic blood pressure, DBP; diastolic blood pressure, BMI; body mass index, WC; waist circumference, Hb; hemoglobin, FBG; fasting blood glucose, Cr; creatinine, eGFR; estimated glomerular filtration rate, AST; aspartate aminotransferase, ALT; alanine aminotransferase, GGT; gamma-glutamyl Transferase, TC; total cholesterol, HDL-C; high-density lipoprotein cholesterol, LDL-C; low-density lipoprotein cholesterol, TG; Triglyceride, SIR; sum of reactogenicity intensity, SIRD; sum of reactogenicity intensity and duration.

**Table 2:** Results of T-spot assay according to the clinical characteristics.

		T-Spot Negative	T-Spot Positive	Total	*p*
Sex	Female	35 (61.4%)	22 (38.6%)	57	0.14
	Male	7 (41.2%)	10 (58.8%)	17	
Age	(years)	35.5 ± 6.7	37.4 ± 5.8	36.8 ± 7.0	0.18
Occupation	Doctor	3 (50.0%)	3 (50.0%)	6	0.45
	Nurse	25 (58.1%)	18 (41.9%)	43	
	Researchers	1 (50%)	1 (50%)	2	
	Medical technologist	7 (43.8%)	9 (56.3%)	16	
	Hospital administrative assistant	6 (85.7%)	1 (14.3%)	7	
Physical activities	Lack	22 (53.7%)	19 (46.3%)	41	0.55
	Adequate	20 (60.6%)	13 (39.4%)	33	
Weight training	Lack	31 (60.8%)	20 (39.2%)	51	0.30
	Adequate	11 (47.8%)	12 (52.2%)	23	
Smoking	No	38 (57.6%)	28 (42.4%)	66	0.68
	Yes	4 (50%)	4 (50%)	8	
Alcohol consumption	Mild to moderate	26 (60.5%)	17 (39.5%)	43	0.45
	Heavy	16 (51.6%)	15 (48.4%)	31	
Anthropometrics	BMI	23.4 ± 3.5	23.2 ± 3.2	23.7 ± 5.0	0.72
	WC	74.7 ± 9.3	73.8 ± 15.6	74.7 ± 12.1	0.76
	SBP	114.26 ± 10.6	117.6 ± 13.7	115.7 ± 11.9	0.25
	DBP	73.1 ± 8.7	75.6 ± 9.2	74.2 ± 9.2	0.24
Pulmonary tuberculosis	No	40 (57.1%)	30 (42.9%)	70	0.78
	Yes	2 (50.0%)	2 (50.0%)	4	
Hypertension	No	40 (56.3%)	31 (43.7%)	71	0.74
	Yes	2 (66.7%)	1 (33.3%)	3	
Dyslipidemia	No	41 (57.7%)	30 (42.3%)	71	0.40
	Yes	1 (33.3%)	2 (66.7%)	3	
Diabetes mellitus	No	41 (56.2%)	32 (43.8%)	73	0.38
	Yes	1 (100.0)	0 (0.0%)	1	
Laboratory analysis	Hb (g/dL)	13.4 ± 1.3	13.8 ± 1.3	13.6 ± 1.3	0.29
	FBG (mg/dL)	94.9 ± 11.8	93.2 ± 15.3	94.4 ± 13.1	0.60
	Cr (mg/dL)	0.6 ± 0.1	0.7 ± 0.2	0.7 ± 0.2	0.01
	eGFR (mL/min/1.73 m^2^)	95.4 ± 19.8	83.8 ± 16.7	90.6 ± 19.5	<0.01
	AST (IU/L)	20.5 ± 5.7	24.8 ± 17.7	22.3 ± 12.1	0.14
	ALT (IU/L)	19.1 ± 14.8	28.1 ± 42.7	22.8 ± 29.5	0.21
	GGT (IU/L)	19.4 ± 13.5	22.7 ± 21.6	20.8 ± 17.0	0.42
	TC (mg/dL)	205.7 ± 36.6	204.1 ± 43.6	205.4 ± 38.8	0.87
	HDL-C (mg/dL)	61.5 ± 10.4	59.0 ± 9.7	60.2 ± 10.1	0.30
	LDL-C (mg/dL)	120.5 ± 35.4	122.1 ± 35.3	121.4 ± 35.0	0.86
	TG (mg/dL)	118.3 ± 72.8	117.6 ± 72.0	118.6 ± 70.4	0.97
Reactogenicity	SIR	14.5 ± 10.0	13.4 ± 8.4	13.4 ± 9.3	0.61
	SIRD	38.1 ± 31.3	34.0 ± 32.9	34.4 ± 31.7	0.58

**Table 3:** Factors associated with the value of anti-S1 antibody, sVNT and T-spot assay using a multivariate linear regression analysis.

	Anti-S1 Antibody				sVNT				T-Spot			
	Univariate Model		Multivariate Model		Univariate Model		Multivariate Model		Univariate Model		Multivariate Model	
	Coefficient (SE)	*p*	Coefficient (SE)	*p*	Coefficient (SE)	*p*	Coefficient (SE)	*p*	Coefficient (SE)	*p*	Coefficient (SE)	*p*
Men	−0.20(0.51)	0.7			−1.78(6.28)	0.77			3.63 (2.07)	0.08	5.94 (2.24)	0.01
Age	−0.07(0.03)	0.02	−0.06(0.03)	0.02	−0.60(0.34)	0.09	−0.61(0.31)	0.05	0.16 (0.14)	0.27		
BMI	0.10(0.06)	0.12	0.10(0.05)	0.06	0.42(0.73)	0.57			−0.32 (0.27)	0.24	−0.69 (0.29)	0.02
Muscle exercise	0.02(0.42)	0.97			3.25(5.07)	0.52			3.30 (1.88)	0.09		
SRI	−0.04(0.03)	0.22			0.19(0.41)	0.65			0.08 (0.04)	0.59	−0.25 (0.15)	0.11
SRID	0.01(0.01)	0.52			−0.08(0.12)	0.50			−0.13 (0.06)	0.89	0.07 (0.04)	0.10

BMI; body mass index, SRI; sum of reactogenicity intensity, SRID; sum of reactogenicity intensity and duration, SE; standard error. Multivariate model selected the variables using backward elimination process.

## Data Availability

The data that support the findings of this study are available on reasonable request from the corresponding author.

## Appendix A

**Table A1:** The association between immunogenicity and experienced type of adverse event according to first and second dose.

		Anti-S1 Antibody (S/co)			sVNT (%)			T-Spot (/250,000 PMCs)		
		Positive Symptom	Negative Symptom	*p*	Positive Symptom	Negative Symptom	*p*	Positive Symptom	Negative Symptom	*p*
1st dose	Fever (58.2%)	5.2 (±1.5)	5.6 (±1.8)	0.26	74.9 (±17.7)	75.1 (±21.4)	0.97	10.4 (±8.1)	9.7 (±7.0)	0.69
	Chills (62.0%)	5.2 (±1.5)	5.67 (±1.81)	0.17	74.0 (±18.3)	76.6 (±30.8)	0.60	10.8 (±1.6)	9.1 (±7.1)	0.34
	Headache (54.4%)	5.1 (±1.3)	5.61 (±1.9)	0.20	74.9 (±17.6)	75.0 (±21.2)	0.97	10.5 (±7.9)	9.7 (±7.4)	0.68
	Muscle pain (72.2%)	5.3 (±1.4)	5.52 (±2.11)	0.62	76.0 (±17.6)	72.2 (±23.0)	0.49	9.9 (±7.1)	10.9 (±9.1)	0.59
	Fatigue (79.7%)	5.3 (±1.4)	5.6 (±2.32)	0.61	75.6 (±17.7)	72.6 (±24.8)	0.66	10.4 (±7.9)	8.9 (±6.2)	0.51
	Nausea & Vomiting (12.7%)	5.9 (±1.4)	5.27 (±1.64)	0.27	80.8 (±14.8)	74.1 (±19.7)	0.31	14.4 (±11.1)	9.5 (±6.8)	0.21
	Rash (5.1%)	6.0 (±0.8)	5.31 (±1.64)	0.41	89.4 (±7.7)	74.2 (±19.3)	0.02	13.9 (±15.3)	9.9 (±7.1)	0.64
	Anaphylactoid reaction (1.3%)	N/A	N/A	N/A	N/A	N/A	N/A	N/A	N/A)	N/A
	Tenderness (84.8%)	5.5 (±1.6)	4.72 (±1.91)	0.15	76.5 (±17.9)	66.4 (±24.4)	0.09	10.4 (±8)	9.0 (±5.4)	0.58
	Pain (53.2%)	10.2 (±8.5)	10.08 (±6.54)	0.32	74.1 (±18.5)	75.9 (±20.2)	0.68	10.2 (±1.3)	10.1 (±1.1)	0.95
	Redness (20.3%)	5.1 (±1.1)	5.4 (±1.74)	0.42	72.4 (±17.3)	75.6 (±19.7)	0.55	7.8 (±5.0)	10.7 (±8.1)	0.19
	Swelling (30.4%)	5.3 (±1.2)	5.38 (±1.77)	0.73	73.6 (±17.0)	75.5 (±20.2)	0.66	8.5 (±7.0)	10.9 (±7.8)	0.20
2nd dose	Fever (20.3%)	5.4 (±1.9)	5.32 (±1.55)	0.81	76.1 (±20.3)	74.7 (±19.1)	0.79	10.2 (±10)	10.1 (±7.0)	0.96
	Chills (20.3%)	6.2 (±1.7)	5.16 (±1.55)	0.03	85.1 (±14.7)	72.8 (±19.4)	0.03	13.0 (±9.6)	9.6 (±7.1)	0.15
	Headache (36.7%)	5.1 (±1.5)	5.47 (±1.71)	0.37	75.4 (±17.8)	74.7 (±20.1)	0.88	11.4 (±6.9)	9.3 (±6.6)	0.27
	Muscle pain (43.0%)	5.5 (±1.6)	5.21 (±1.65)	0.41	78.0 (±17.7)	72.7 (±20.1)	0.23	10.2 (±8.9)	10.1 (±6.6)	0.98
	Fatigue (59.5%)	5.4 (±1.5)	5.26 (±1.85)	0.71	77.6 (±16.5)	71.0 (±22.2)	0.16	10.8 (±8.4)	9.1 (±6.2)	0.37
	Nausea & Vomiting (3.8%)	6.1 (±1.3)	5.31 (±1.63)	0.40	83.7 (±14.1)	74.6 (±19.4)	0.43	16.3 (±16)	9.9 (±7.2)	0.56
	Rash (1.3%)	N/A	N/A	N/A	N/A	N/A	N/A	N/A	N/A	N/A
	Anaphylactoid reaction (0.0%)	N/A	N/A)	N/A	N/A	N/A	N/A	N/A	N/A	N/A
	Tenderness (67.1%)	5.4 (±1.5)	5.18 (±1.88)	0.52	77.8 (±16.4)	69.3 (±23.3)	0.10	9.5 (±7.3)	11.5 (±8.3)	0.29
	Pain (36.7%)	5.3 (±1.5)	5.39 (±1.7)	0.72	75.1 (±17.9)	74.9 (±20.1)	0.97	8.7 (±7.4)	11.0 (±7.7)	0.22
	Redness (17.7%)	5.1 (±1.3)	5.4 (±1.68)	0.48	71.2 (±18.4)	75.8 (±19.4)	0.42	7.8 (±5.4)	10.6 (±8.0)	0.23
	Swelling (16.5%)	5.1 (±1.1)	5.38 (±1.71)	0.63	71.2 (±16.3)	75.7 (±19.7)	0.44	7.0 (±5.2)	10.7 (±7.9)	0.12

Data are presented as mean ± standard deviation for continuous variables. S/Co; signal to cut-off, sVNT; surrogate virus neutralization test.

**Table A2:** The association between immunogenicity and individual’s health state, antipyretics, and reactogenicity index.

			Anti-S1 Antibody		sVNT		T-Spot	
	Variables (N)		Mean (SD)	*p*	Mean (SD)	*p*	Mean (SD)	*p*
Sex	Male (21)		5.2 (±1.9)	0.73	73.7 (±20.9)	0.73	12.9 (±7.8)	0.08
	Female (58)		5.4 (±1.5)		75.4 (±18.7)		9.3 (±7.4)	
Age	<40 (55)		5.4 (±1.6)	0.47	76.6 (±17.7)	0.31	9.8 (±7.6)	0.58
	≥40 (24)		5.1 (±1.8)		71.2 (±22.2)		10.9 (±7.7)	
Smoking	No (70)		5.3 (±1.7)	0.26	74.0 (±19.5)	0.23	10.1 (±7.9)	0.83
	Yes (9)		5.9 (±1.3)		82.2 (±16.1)		10.7 (±4.8)	
Alcohol consumption	Mild to moderate (45)		5.3 (±1.5)	0.98	75.5 (±18.6)	0.78	8.9 (±5.9)	0.13
	Heavy or binge (34)		5.3 (±1.7)		74.2 (±20.2)		11.9 (±9.3)	
BMI	<25 (57)		5.2 (±1.5)	0.44	74.2 (±18.3)	0.51	10.1 (±7.9)	0.08
	25–30 (18)		5.7 (±0.4)		78.7 (±19.8)		11.8 (±6.7)	
	>30 (4)		6.1 (±0.8)		68.8 (±31.4)		4.0 (±5.0)	
Pulmonary tuberculosis	No (75)		5.3 (±1.6)	0.43	74.4 (±19.3)	0.27	10.2 (±7.7)	0.92
	Yes(4)		6.0 (±0.8)		85.4 (±15.7)		7.8 (±6.0)	
HTN	No (76)		5.3 (±1.6)	0.90	75.2 (±19.0)	0.50	10.3 (±7.7)	0.47
	Yes (3)		5.5 (±2.8)		67.7 (±26.4)		7.0 (±3.5)	
Dyslipidemia	No (76)		5.3 (±1.6)	0.94	74.7 (±19.0)	0.62	10.1 (±7.7)	0.78
	Yes (3)		5.4 (±1.8)		80.4 (±28.5)		11.3 (±5.8)	
DM	No (78)		5.4 (±1.6)	0.24	75.3 (±19.1)	0.15	10.2 (±7.6)	0.50
	Yes (1)		3.5 (±NA)		47.5 (±NA)		5.0 (±NA)	
Abnormal LFT	No (71)		5.4 (±1.7)	0.90	75.1 (±18.7)	0.84	10.3 (±7.8)	0.67
	Yes (8)		5.3 (±1.4)		73.7 (±24.5)		9.1 (±5.9)	
Abnormal RFT	No (72)		5.3 (±1.6)	0.25	74.7 (±19.5)	0.75	9.6 (±7.0)	0.19
	Yes (7)		6.0 (±1.4)		77.1 (±16.8)		15.7 (±11.0)	
Physical activity	Lack (42)		5.2 (±1.6)	0.52	73.1 (±21.2)	0.36	10.6 (±7.4)	0.60
	Adequate (37)		5.5 (±1.6)		77.0 (±16.7)		9.6 (±7.9)	
Muscle exercise	Lack (52)		5.3 (±1.4)	0.80	74.0 (±18.7)	0.55	9.1 (±7.0)	0.09
	Adequate (27)		5.4 (±1.9)		76.8 (±20.4)		12.4 (±8.6)	
1st dose	Antipyretics	No (8)	5.4 (±1.5)	0.68	76.2 (±17.9)	0.14	10.1 (±7.7)	0.97
		Yes (69)	5.0 (±2.5)		66.5 (±26.1)		10.3 (±7.3)	
	Antipyretics intake	Prophylactic (30)	5.4 (±1.5)	0.58	75.1 (±17.5)	0.62	10.5 (±7.4)	0.76
		After symptom onset (39)	5.4 (±1.5)		77.4 (±18.4)		9.9 (±8.1)	
2nd dose	Antipyretics	No (28)	5.4 (±1.5)	0.83	76.1 (±17.6)	0.46	10.6 (±8.4)	0.51
		Yes (51)	5.3 (±1.8)		72.8 (±21.9)		9.3 (±5.7)	
	Antipyretics intake	Prophylactic (24)	5.5 (±1.8)	0.51	76.3 (±17.2)	0.85	11.9 (±8.9)	0.33
		After symptom onset (27)	5.2 (±1.3)		75.4 (±18.3)		9.5 (±8.3)	
SRI	<13 (37)		5.3 (±1.8)	0.81	72.9 (±21.0)	0.38	10.8 (±7.8)	0.49
	≥13 (42)		5.4 (±1.5)		76.8 (±17.5)		9.6 (±7.5)	
SRID	<29 (40)		5.4 (±1.8)	0.75	74.6 (±20.8)	0.88	11.3 (±7.7)	0.21
	≥29 (39)		5.3 (±1.5)		75.3 (±17.6)		9.1 (±7.5)	

BMI; body mass index, HTN; hypertension, DM; diabetes mellitus, LFT; liver function test, RFT; renal function test. Data are presented as mean ± standard deviation for continuous variables. S/Co; signal to cut-off, sVNT; surrogate virus neutralization test.
